# Cutting Staff Radiation Exposure and Improving Freedom of Motion during CT Interventions: Comparison of a Novel Workflow Utilizing a Radiation Protection Cabin versus Two Conventional Workflows

**DOI:** 10.3390/diagnostics11061099

**Published:** 2021-06-16

**Authors:** Peter Dankerl, Matthias Stefan May, Christian Canstein, Michael Uder, Marc Saake

**Affiliations:** 1 Department of Radiology, University Hospital Erlangen, Maximiliansplatz 3, 91054 Erlangen, Germany; matthias.may@uk-erlangen.de (M.S.M.); michael.uder@uk-erlangen.de (M.U.); marc.saake@uk-erlangen.de (M.S.); 2 Siemens Healthcare GmbH, Siemensstr. 3, 91031 Forchheim, Germany; christian.canstein@siemens-healthineers.com

**Keywords:** radiation exposure, CT intervention, radiation protection, medical staff dose, lead apron, workflow

## Abstract

This study aimed to evaluate the radiation exposure to the radiologist and the procedure time of prospectively matched CT interventions implementing three different workflows—the radiologist—(I) leaving the CT room during scanning; (II) wearing a lead apron and staying in the CT room; (III) staying in the CT room in a prototype radiation protection cabin without lead apron while utilizing a wireless remote control and a tablet. We prospectively evaluated the radiologist’s radiation exposure utilizing an electronic personal dosimeter, the intervention time, and success in CT interventions matched to the three different workflows. We compared the interventional success, the patient’s dose of the interventional scans in each workflow (total mAs and total DLP), the radiologist’s personal dose (in µSV), and interventional time. To perform workflow III, a prototype of a radiation protection cabin, with 3 mm lead equivalent walls and a foot switch to operate the doors, was built in the CT examination room. Radiation exposure during the maximum tube output at 120 kV was measured by the local admission officials inside the cabin at the same level as in the technician’s control room (below 0.5 μSv/h and 1 mSv/y). Further, to utilize the full potential of this novel workflow, a sterile packed remote control (to move the CT table and to trigger the radiation) and a sterile packed tablet anchored on the CT table (to plan and navigate during the CT intervention) were operated by the radiologist. There were 18 interventions performed in workflow I, 16 in workflow II, and 27 in workflow III. There were no significant differences in the intervention time (workflow I: 23 min ± 12, workflow II: 20 min ± 8, and workflow III: 21 min ± 10, *p* = 0.71) and the patient’s dose (total DLP, *p* = 0.14). However, the personal dosimeter registered 0.17 ± 0.22 µSv for workflow II, while I and III both documented 0 µSv, displaying significant difference (*p* < 0.001). All workflows were performed completely and successfully in all cases. The new workflow has the potential to reduce interventional CT radiologists’ radiation dose to zero while relieving them from working in a lead apron all day.

## 1. Introduction

Image-guided percutaneous needle biopsy is the gold standard to probe and diagnose most types of cancer [1]. While superficial lesions and lymph nodes can often be probed utilizing sonography, CT-guided biopsy to date is the most frequently applied method of choice. It has been deployed since 1976 when the first lung biopsy utilizing CT guidance was performed [2]. Five years later, the first spine lesion was probed via CT guidance [3]. Another 15 years later, real-time CT fluoroscopy was introduced into clinical practice [4] and has been shown to reduce interventional procedure times but has increased radiation exposure for the patient and the interventionalist [5]. Therefore, the conventional CT-guided biopsy (without fluoroscopy) remains the predominant workflow in clinical practice, even in lung biopsies [6].

However, the conventional and the CT-fluoroscopic workflows both comprise some disadvantages. During the conventional workflow, the radiologist advances the needle position, steps out of the CT room to scan, and localizes the needle tip and target repeatedly until reaching the target position [5]. For the fluoroscopic procedure, the radiologist stays in the CT room and wears a lead apron as is exposed to X-ray radiation. This can lead to a substantial cumulative occupational dose in interventionalists—with still unknown long-term effects on health [7]. In order to combine the benefits of both workflows—the procedural speed and close contact to the patient of the fluoroscopic workflow with the staff’s radiation protection and freedom of motion (due to the gratuitous lead apron) of the conventional workflow, we set up a radiation protection cabin inside the CT room and investigated the so-called “mobile workflow” [8]. This cabin features 3 mm lead equivalent walls and a foot switch to operate the doors. To further speed up the interventional procedure, the radiologists utilize a sterile packed remote control to move the CT table and trigger the scan and a sterile packed tablet PC anchored on the CT table to view images, plan trajectory, and navigate during the CT intervention.

The aim of this study was to evaluate the radiation exposure, procedure time, and success of CT interventions implementing the conventional workflow with the radiologist leaving the CT room, a CT fluoroscopic like workflow where the internationalist wears a lead apron and stays in the CT room, and the mobile workflow utilizing the radiation protection cabin, remote control, and a tablet PC while staying inside the CT room.

We hypothesize that the mobile workflow exposes the radiologist to significantly less radiation than the fluoroscopic-like workflow while being comparatively speedy.

## 2. Materials and Methods

This study was conducted in accordance with the guidelines of the Declaration of Helsinki and approved by the Ethics Committee of University Hospital Erlangen under the approval number 258_18B. The Ethics Committee waived the written informed consent requirement.

We prospectively evaluated the radiologist’s radiation exposure utilizing an electronic personal dosimeter and the intervention time in CT interventions matched to the three different workflows. We compared the dose of the interventional scans in each workflow (total mAs and total DLP), the radiologist’s personal dose (in µSV), and the interventional time (in sec).

To perform workflow III, a prototype of a radiation protection cabin with 3 mm lead equivalent walls and a foot switch to operate the doors was built in the CT examination room. Radiation exposure during the maximum tube output at 120 kV was measured by the local admission officials at the same level as in the technician’s control room (<0.5 μSv/h and <1 mSv/y). Further, to utilize the full potential of the mobile workflow, a sterile packed remote control (to move the CT table and to trigger the radiation), and a sterile packed tablet PC anchored on the CT table (to plan and navigate during the CT intervention) were operated by the radiologist.

### 2.1. Patient Population

In this study, 61 consecutive patients who received CT interventions were prospectively matched to three different groups comprising of the three different workflows. The matching criteria were in accordance with the STROBE guidelines [9], where the different types of CT interventions consisted of shoulder/hip arthrography, core needle biopsy in accordance with the organ/body region (lung, mediastinum, liver, pancreas, retroperitoneal, mesenterial, kidney, bone), and drainage catheter placement in accordance with the body region. Further, we matched as per the patient’s age.

In group/workflow I, we had 18 patients, 6 with arthrography and 12 with biopsies, with a mean age of 54 ± 21 years; in group/workflow II, we had 16 patients, 4 with arthrography, 4 receiving drainage catheter, and 8 with biopsies, with a mean age of 57 ± 21 years; in group/workflow III, we had 27 patients, 6 with arthrography, 5 receiving drainage catheter, and 16 with biopsies with a mean age of 54 ± 17 years.

### 2.2. CT Intervention Workflows

All interventions were performed utilizing a 128-slice SOMATOM go.Top CT scanner (Siemens Healthineers, Erlangen, Germany). In all three workflows, the patients were first placed on the CT table in either prone or supine position and a spiral scan was performed of the body region where the lesion had been detected in a previous examination. Within this dataset, the lesion was located, and the interventional path was planned by utilizing the Guide&GO intervention tool (Siemens Healthineers). The table was moved to the corresponding position of the planned puncture site and utilizing the laser light in the gantry, the estimated skin location was marked with a radio-opaque target. To verify the skin marking, a single slice CT scan was performed, and if the marked skin location corresponded with the planned needle pathway, a sterile drape was placed, the skin was disinfected, and local anesthetics were injected into this location; if the radio-opaque target and the previously planned path did not match, target repositioning and single slice scanning were performed until satisfaction.

In workflow I, the interventional radiologist put on a surgical gown and stepwise advanced the biopsy/drainage needle towards the desired location. After every manipulation of the needle position, the radiologist left the CT room and the assisting technician performed either a sequential or spiral scan in regard to interventional needs. This was repeated until the lesion was hit with the needle. In case of a drainage catheter placement via Seldinger’s maneuver, the location of the wire tip needs to be observed during the procedure. To do this without staying in the CT room, we usually fix the part of the guidewire outside the patient’s body to the surgical drape using a sterile clamp during the CT scan. For additional protection against contamination (e.g., from contact to the gantry during scans), we leave this part of the guidewire inside the protective sheath it comes with.

In workflow II, the interventional radiologist put on a wrap-around lead apron with a 0.7 mm lead equivalent on the front and 0.35 mm on the back and a thyroid shield with 0.5 mm lead equivalent under the surgical gown. To observe the needle position during the intervention, the radiologist stayed in the CT room and operated the CT table positioning and scanning while standing in the radiation shadow next to the gantry. We call this workflow the fluoroscopic-like workflow as the utilized scan modes were either sequential or spiral and not fluoroscopic as institutional best practice guidelines recommend relinquishing fluoroscopic needle advancement as often as possible. Only during some stages of interventions, the interventionalist stood next to the patient for a control scan (most frequently in sequential mode). For example, in some drainage catheter placements via Seldinger’s maneuver, the established wire tip location was verified by manually holding the wire in position during the control scan. In previous work on intermittent fluoroscopy [10], a foot switch and table-mounted control panel have been used for scanning and table positioning. However, in workflow II, the so-called fluoroscopic-like workflow, we instead utilized a wireless remote control that was placed in a sterile cover and featured a safety lock system to not accidentally trigger radiation. Furthermore, this procedure was repeated until the lesion was hit with the needle.

**Figure 1:**
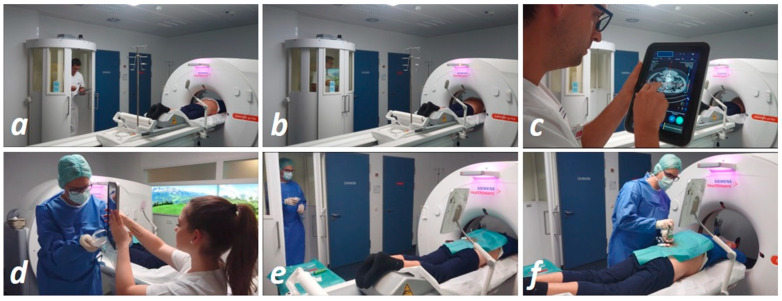
Procedure of the novel mobile workflow: (**a**) tablet PC and remote control are used to set up for initial scanning; (**b**) doors of the radiation protection cabin are closed via the foot switch during scanning; (**c**) planning of the needle path is performed on the touchscreen of the tablet; (**d**) for the aseptic part of the intervention, the remote control and tablet PC are packed sterile; (**e**) during the stepwise needle placement, scanning is performed from the radiation protection cabin by the radiologist; (**f**) biopsy of the lesion.

In workflow III, the interventional radiologist put on a surgical gown and stepwise advanced the biopsy/drainage needle towards the desired location. After every manipulation of the needle position, the radiologist stepped into the radiation protection cabin next to the CT table, closed the cabin doors using a foot switch, and initiated a CT scan utilizing the wireless remote control. From inside the radiation protection cabin, the radiologist could talk to the patient (e.g., for breathing commands), while moving the CT table and initiating radiation utilizing the remote control. In accordance with the previous workflows, this procedure was repeated until satisfactory (Figure 1). When performing a drainage catheter placement via Seldinger’s maneuver in this workflow, in accordance with workflow I, we clamped the part of the guidewire outside the patient’s body to the surgical drape using a sterile clamp, allowing the radiologist to enter the radiation protection cabin during the CT scan (Supplementary Materials Video S1).

### 2.3. Radiation Protection Cabin

A prototype of a radiation protection cabin with 3 mm lead equivalent walls was built in the CT examination room next to the CT table. In order to remain sterile during the procedure and for repetitive entry and exit of the radiation protection cabin, a foot switch was installed to operate the doors. Furthermore, the size of the protection cabin is large enough and the doors of the cabin open wide enough to allow for sufficient safety distance in all directions while inside the cabin. The radiation protection cabin has an outside dimension of 135 cm width, 80 cm depth, and 220 cm height. Radiation exposure during the maximum tube output at 120 kV was measured by the local admission officials at the same level as in the technician’s control room (0.5 μSv/h) and below 1 mSv/y. Further, to utilize the full potential of the mobile workflow, a sterile packed remote control (to move the CT table and to trigger the radiation) and a sterile packed tablet anchored on the CT table (to plan and navigate during the CT intervention) were operated by the radiologist.

### 2.4. Wireless Remote Control

In order to operate the CT table, a wireless remote control was utilized. To be able to use the remote control under sterile conditions, the remote was packed into a sterile cover. To not accidentally trigger radiation, a safety lock system had been built into the remote control. Only if the interventional radiologist firmly held the remote with one hand, the radiation could be initiated. This prevents an accidental release of radiation, for instance, if the remote control dropped or something on the sterile table touched the radiation button.

### 2.5. Tablet PC

All CT interventions were performed on the Somatom go.Top (Siemens Healthcare GmbH, Forchheim, Germany). Besides being able to operate the CT with a conventional stationary console from the technician’s antechamber, limited operational functions (e.g., registration, planning, examination, automated reconstruction, and automated archiving) of this scanner can be operated by a mobile tablet PC (Elite ×2 1012 G1, Hewlett-Packard Inc., Palo Alto, CA, USA). The tablet is equipped with a 12-inch full high-definition display and a 64-bit operating system (Windows 10 Professional, Microsoft Corp., Redmont, WA, USA). Furthermore, interventional planning or visualization of control scans and derived interactions (image navigation, zooming, panning, windowing) can be performed on the tablet utilizing the touchscreen.

### 2.6. Interventional Radiation Exposure and Procedure Time

**Figure 2:**
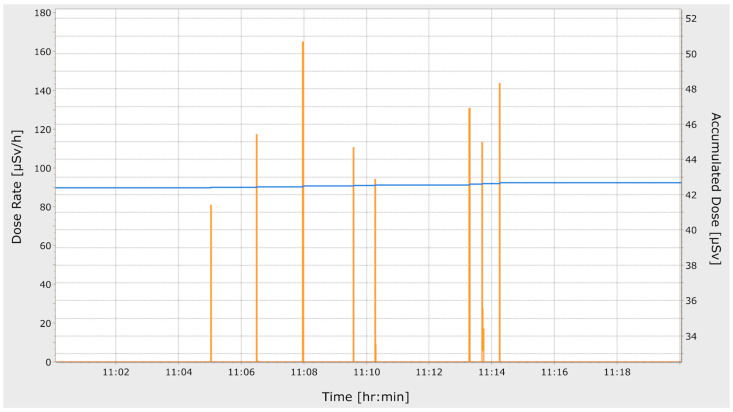
Example readout of the personal dosimeter of an intervention in workflow II (radiologist inside the radiation protection cabin during CT scans). Each red spike represents an interventional scan. The height of the spikes correlates with the radiation dose depending on the position of the radiologist during scanning. The blue line represents the total accumulated dose of the dosimeter in µSv and demonstrates a minimal increase between the first and last scans.

We separately documented the patient’s dose of each interventional scan in all of the three different workflows as volume computed tomography dose index (CTDI_vol_) and as dose length product (DLP). Furthermore, the total mAs and total DLP of the procedures were registered. During all interventions, a personal dosimeter was placed on top of the lead apron/scrubs (depending on the workflow) on the interventionist’s chest and only covered by the sterile gown. The personal dose was documented in µSV utilizing a RaySafe i3 (Unfors RaySafe AB, Billdal, Sweden) personal dosimeter facilitating accurate real-time monitoring (Figure 2) of radiation dose during interventional radiology [11].

Further, for each intervention, start and end times were documented including patient positioning, procedure planning, sterile dressing and draping, application, and reaction time of local anesthetics to the final control scan after the procedure has ended. Further, it was documented if all procedures were performed successfully without switching over to another workflow during the procedures.

### 2.7. Statistical Analysis

Quantitative variables are expressed as a mean ± standard deviation, whereas categorical variables are expressed as frequencies. The data were tested for normal distribution using the Kolmogorov–Smirnov test. Groups were compared using one-way ANOVA for quantitative variables. SPSS 21 (IBM Corporation, Armonk, NY, USA) was used for the analysis. A *p*-value of <0.05 was considered statistically significant. Artworks were generated using SPSS 21 and Photoshop Extended (Version CS6, Adobe Systems, San Jose, CA, USA).

## 3. Results

The detailed results can be found in Table 1.

There were no statistically significant differences between the three workflows considering the procedure time (ANOVA, *p*-value 0.71). When grouping the two workflows with the interventionalist inside the CT room (either in the radiation protection cabin or next to the gantry) and comparing to the workflow where the room is left, we did not find any significant differences (Mann–Whitney U test, *p*-value = 0.63).

Furthermore, after Shapiro–Wilk test demonstrated non-normal distribution for the patient’s radiation dose during the entire examinations (total DLP), we performed Welch’s ANOVA but did not find any significant differences as well (*p* = 0.14).

Kruskal–Wallis test, nevertheless, found significant differences for the interventionalists’ mean personal dose (*p* < 0.001) between the three different workflows.

All workflows were performed completely and successfully in all cases without having to switch into a lead apron in workflow III during the procedure.

## 4. Discussion

In our study, we present a novel interventional workflow utilizing a radiation protection cabin, a sterile tablet PC, and wireless remote control to enable safe and successful CT interventions. We found that this workflow effectively shelters the interventional radiologist from any personal radiation dose without the need to wear a lead apron while staying in close contact with the patient.

As interventional radiologists have been reported to suffer from work-related musculoskeletal symptoms [12,13,14], an interventional workflow without the need for lead aprons appears to be beneficial and welcome. Nevertheless, especially for tricky interventional procedures (e.g., the biopsy of small lung nodules) with close patient contact, the possibility of direct patient interaction (for instance, breathing instructions), and a fast operation without leaving the CT room, can improve procedural comfort and success. All these features are met in the presented workflow using the radiation protection cabin set-up right next to the CT table. In contrast to previous work, the cabin does not have to be wrapped in a sterile drape kit [15] and—even more important—shielded the interventionalist from any measurable radiation exposure [15,16]. Considering that age significantly affects the lifetime-attributed risk and excess relative risk of cancer mortality [17], medical staff’s radiation protection cannot be regarded highly enough during CT interventions. However, the mobile workflow does not affect the radiation dose the patient receives during the procedure. In order to reduce the radiation dose for the patient, tin filtration [18], low-dose examination protocols [19,20] or optical needle tracking devices [21], robotic guidance [22], or even augmented reality [23] could be utilized during interventional procedures.

Although not statistically significant, in accordance with previous research [24], a trend towards shorter intervention times could be documented for the presented novel workflow, in contrast to the conventional workflow where the radiologist leaves the CT room. The fluoroscopic workflow offers valuable benefits compared to the conventional workflow, such as a reduced need for sedation/analgesia, reduced costs, and even lower complications [25,26]. We believe the presented mobile workflow combines these benefits with the advantage of erased radiation exposure to the interventionalist.

Although future advances in nuclear medicine [27] and liquid biopsy [28] could potentially make image-guided tissue biopsy expendable, to date, a biopsy of suspicious lesions is the gold standard in the diagnostic workup of cancer and its differential diagnosis. As demonstrated by Guberina et al. [29], radiation exposure during CT interventions is largely dependent on the scanner generation. However, as demonstrated by our novel mobile workflow, and in accordance with the request by Leng [30], by implementing a radiation protection cabin into the interventional procedure, radiation exposure can be drastically reduced for the medical staff.

One limitation of our experimental setup was the fact that the personal dosimeter was worn on top of the lead apron underneath the sterile gown in workflow II, while it was worn on top of the scrubs underneath the sterile gown in workflows I and III. Therefore, formally we did not measure the exposure to the radiologist, but the exposure of the protective equipment. We conducted a pre-study in which we placed the dosimeter under the protective equipment. However, with this setup, the residual dose penetrating the lead protection and reaching the dosimeter was below the lower measurement limit of the dosimeter. Therefore, we decided to place the dosimeter over the protective equipment. Considering that the relative dose shielding effect of the lead protection is constant (for a constant X-ray spectrum, e.g., 90%) the radiologist’s personal dose can be estimated from the data. Importantly, in the proposed new workflow III, the radiologist’s dose without a lead apron is below the lower measurement limit.

A further limitation of our study is that it is a single-center evaluation consisting of a moderate amount of CT interventions in the respective groups. Although matched, the types of intervention are not perfectly leveled between the three groups. The personal dosimeter used might have missed minor irradiation below the detection limit (<30 μSv/h). Moreover, in some complicated interventions, the hand of the radiologist might be needed at the material during the whole procedure. However, during most procedures in our institute, the radiologist leaves the room or stands beside the CT scanner during scanning without holding onto the material to reduce the radiologist’s dose.

## 5. Conclusions

The presented novel workflow enables safe and successful CT interventions in a time period comparable to the conventional workflows, but without the burden of wearing a lead apron and without any measurable radiation exposure to the interventionalist.

## Figures and Tables

**Table 1:** Demographic details and study results. Workflows: The radiologist—(I) leaving the CT room during scanning; (II) wearing a lead apron and staying in the CT room; (III) staying in the CT room in a prototype radiation protection cabin without lead apron while utilizing a wireless remote control and a tablet. Numbers are given as mean ± standard deviation. A *p*-value < 0.05 was considered statistically significant and marked with an asterisk.

	Workflow			*p*
	I	II	III	
Number of patients	18	16	27	
Age of patients (in years)	54 ± 21	57 ± 21	54 ± 17	
Total DLP	355 ± 318	339 ± 263	484 ± 689	0.14
Interventional procedure time (in min)	23 ± 12	20 ± 8	21 ± 10	0.71
Number of scans	6 ± 3	6 ± 2	6 ± 3	
Measured effective dose (in µSv)	0 ± 0	0.17 ± 0.22	0 ± 0	<0.001 *

## Data Availability

Data available on request due to restrictions e.g., privacy or ethical. The data presented in this study are available on request from the corresponding author. The data are not publicly available due to privacy.

## Supplementary Materials

The following are available online at https://www.mdpi.com/article/10.3390/diagnostics11061099/s1, Video S1: Condensed video of investigated workflow III featuring the clinical use of the radiation protection cabin, the wireless remote control and the tablet.
